# Research and Implementation of Autonomous Navigation for Mobile Robots Based on SLAM Algorithm under ROS

**DOI:** 10.3390/s22114172

**Published:** 2022-05-31

**Authors:** Jianwei Zhao, Shengyi Liu, Jinyu Li

**Affiliations:** 1 School of Mechanical Electronic & Information Engineering, China University of Mining and Technology, Beijing 100089, China; zhaojianwei@cumtb.edu.cn (J.Z.); sqt2100402034@student.cumtb.edu.cn (J.L.); 2 Institute of Artificial Intelligence, University of Science and Technology Beijing, Beijing 100083, China

**Keywords:** SLAM, ROS, A* algorithm, autonomous navigation

## Abstract

Aiming at the problems of low mapping accuracy, slow path planning efficiency, and high radar frequency requirements in the process of mobile robot mapping and navigation in an indoor environment, this paper proposes a four-wheel drive adaptive robot positioning and navigation system based on ROS. By comparing and analyzing the mapping effects of various 2D-SLAM algorithms (Gmapping, Karto SLAM, and Hector SLAM), the Karto SLAM algorithm is used for map building. By comparing the Dijkstra algorithm with the A* algorithm, the A* algorithm is used for heuristic searches, which improves the efficiency of path planning. The DWA algorithm is used for local path planning, and real-time path planning is carried out by combining sensor data, which have a good obstacle avoidance performance. The mathematical model of four-wheel adaptive robot sliding steering was established, and the URDF model of the mobile robot was established under a ROS system. The map environment was built in Gazebo, and the simulation experiment was carried out by integrating lidar and odometer data, so as to realize the functions of mobile robot scanning mapping and autonomous obstacle avoidance navigation. The communication between the ROS system and STM32 is realized, the packaging of the ROS chassis node is completed, and the ROS chassis node has the function of receiving speed commands and feeding back odometer data and TF transformation, and the slip rate of the four-wheel robot in situ steering is successfully measured, making the chassis pose more accurate. Simulation tests and experimental verification show that the system has a high precision in environment map building and can achieve accurate navigation tasks.

## 1. Introduction

With the continuous development of the advanced manufacturing industry and artificial intelligence, intelligent automatic operation has gradually replaced the traditional cooperative operation mode of mechanical production and manual supervision. The application of robots is gradually infiltrating into all fields of our life and forming a multi-cross and multi-integrated discipline [1]. With the continuous development of advanced algorithms and sensor technology, mobile robots with an autonomous navigation ability are gradually applied in indoor and outdoor locations [2]. A robot capable of autonomous mapping and navigation needs to have the ability of environmental perception, positioning, and path planning [3]. Liao Maosheng used the indoor mobile robot platform based on raspberry PI to construct indoor maps with the Gmapping algorithm, and the results showed the robustness of navigation [4]. Wang Peng used the Gmapping algorithm for map building and adopted an A* algorithm and a DWA (Dynamic Windows Approach) algorithm for path planning. By introducing the evaluation function of global information, the problem of falling into a local optimum is solved. However, there are still problems of dependence on an odometer and a low accuracy of map construction [5]. Shou Jiaxin chose a Hector algorithm for map construction and the Dijkstra algorithm for path planning, which reduced the dependence on the odometer, but there were still problems such as high requirements on lidar frequency and a slow path search efficiency [6].

Aiming at the problems of a low mapping accuracy and a high lidar frequency requirement of the robot navigation system, this paper proposes a mobile robot mapping and navigation system based on a ROS (Robot Operating System). Through the simulation analysis of various indoor SLAM algorithms, the Karto SLAM algorithm with a high robustness and loopback detection is selected for map construction to improve the accuracy of the map construction, and A* and DWA algorithms are selected for path planning to improve the efficiency of the path search. Through simulation and practical verification, the system can not only accurately locate and build an environmental map, but also achieve accurate navigation.

## 2. Architecture Design of Four-Wheel Adaptive Robot System

### 2.1. Overall System Design

**Figure 1:**
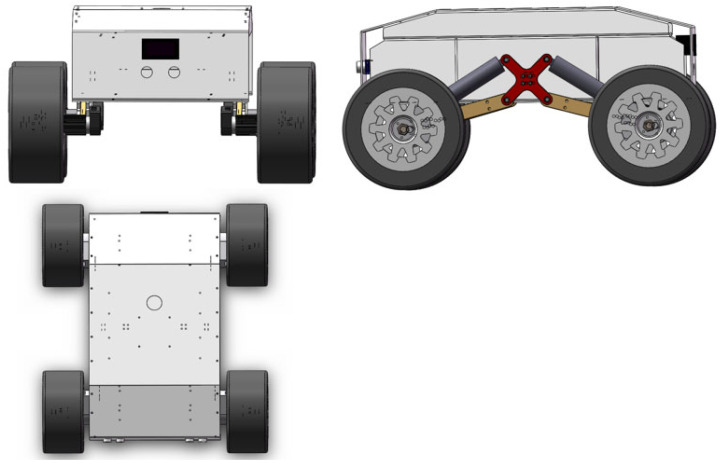
Four-wheel adaptive robot model.

The four-wheel adaptive robot model used in this paper is shown in Figure 1. The wheels and the body are connected by a spring suspension, which can realize SLAM and autonomous navigation in various complex terrains

The main components and models of the robot are shown in Table 1.

**Figure 2:**
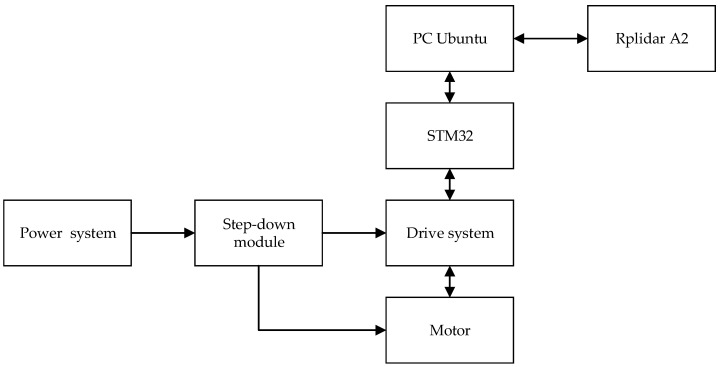
Robot hardware connection diagram.

The hardware connection relation of each module is shown in Figure 2.

### 2.2. Analysis of Adaptive Damping Mechanism

The car body adopts an enclosed design to reduce the risk of an easy loss of exposed components. Considering the vehicle structure and operating environment, the spring damping system is adopted for damping. It reduces body vibration and prevents tires from losing grip.

By analyzing the spring, the damping buffer force of the spring can be obtained:

(1) $$F_{t}=K\times (L_{0}-l_{0})=267\times [(120-22.5)/1000]=26.03N$$

where *K* is the spring coefficient, *L*_0_ is the initial spring length, and *l*_0_ is the shortest spring length.

**Figure 3:**
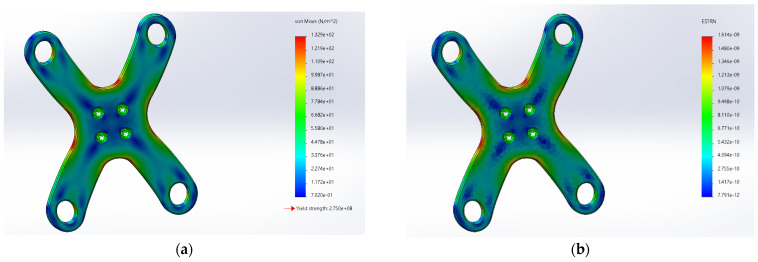
SolidWorks Simulation diagram. (**a**) Static stress figure; (**b**) strain figure.

The “X” arm was imported in SolidWorks Simulation. Material selection, constraint setting, pressure application, and meshing were carried out for the “X” arm, and the static stress analysis and strain cloud images were obtained through calculation as shown in Figure 3.

Through analysis and calculation, it can be seen that the overall force of the “X” arm is relatively uniform after the external load is applied, and most of the arm is in the blue area of the stress cloud. The stress at the intersection is more concentrated, but also within the yield strength. The overall deformation of the parts is small, and the deformation is relatively large at the intersection, but it is within the safe range of strain and meets the strength requirement.

### 2.3. Adaptive Chassis Steering Analysis

In the design of the mobile robot chassis, the performance of the hub structure directly determines the robot’s moving and steering mode. In a four-wheel robot chassis, commonly used steering methods include Ackerman steering, waist joint steering, and sliding steering. According to the driving mode, the four-wheel sliding steering robot can be divided into three kinds: left and right two-wheel unified drive, front two-wheel or rear two-wheel drive, and four-wheel independent drive. The robot chassis used in this paper is equipped with four driving motors, which are independent of four wheels. 

**Figure 4:**
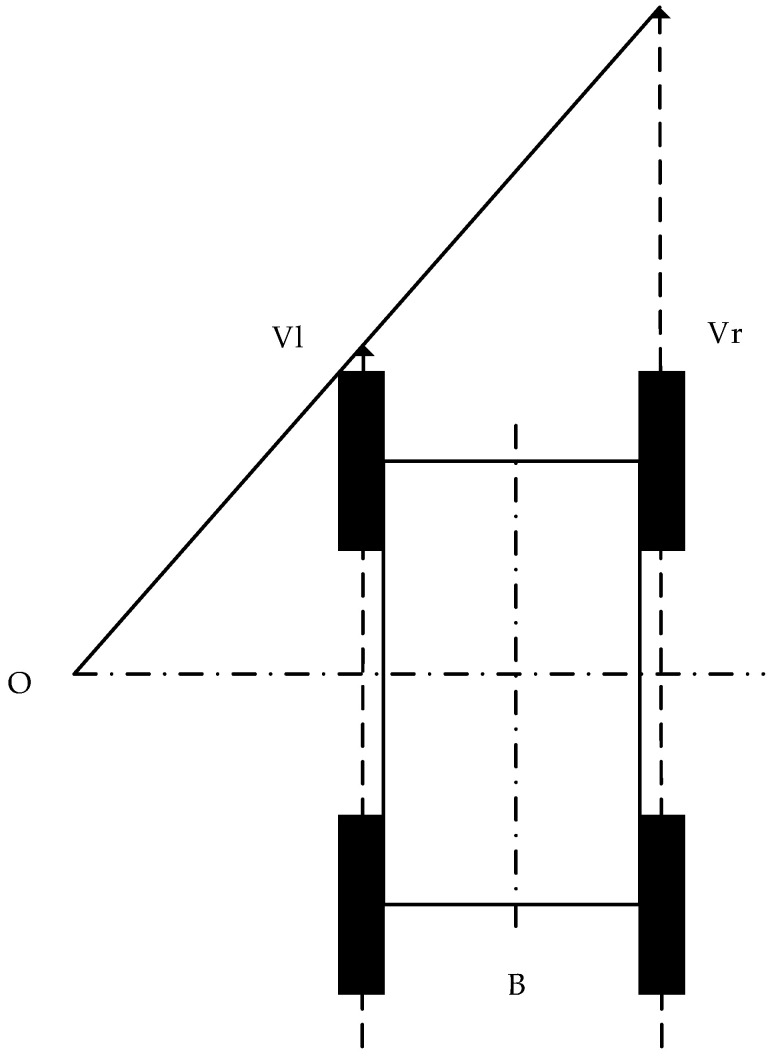
Structure of four-wheel slip-steering robot.

Due to the rules of the car body structure, the symmetrical distribution of the wheel hubs, and an indoor environment, there are the following assumptions in the construction of the model: the car’s center of mass coincides with its geometric center; when turning, the motion center and the longitudinal symmetry center are located in the same horizontal plane, the longitudinal force on the same side wheel is equal [7], and the rotation speed is the same; the robot is always moving in a plane; and the four-wheel robot with the same speed on the same side is similar to the tracked vehicle, and its structure is shown in Figure 4.

If the left wheel speed is *V_l_*, the right wheel speed is *V_r_*, and the body width is *B* then the linear velocity and angular velocity of the robot are:

(2) $$\{\begin{array}{c} V=(V_{l}+V_{r})/2\\ \omega =(V_{r}-V_{l})/B \end{array}$$

In the actual steering, due to the different rotation speeds of the inner and outer wheels, the linear speed of the outer wheel is greater than that of the robot, while the linear speed of the inner wheel is less than that of the robot, resulting in the relative sliding of the wheels on both sides, and the actual wheel speed changes. The left wheel speed is *n*_1_, the right wheel speed is *n*_2_, the wheel radius is *r*, and the slip rate of the left- and right-side wheels is defined as:

(3) $$\{\begin{array}{c} \mu_{l}=\frac{n_{1}r-V_{l}}{n_{1}r}\\ \mu_{r}=\frac{n_{2}r-V_{r}}{n_{2}r} \end{array}$$

When the average slip rate of the left and right wheels is known, the actual wheel speed on the left and right sides can be expressed as:

(4) $$\{\begin{array}{c} V_{l}=n_{1}r(1-\mu_{l})\\ V_{r}=n_{2}r(1-\mu_{2}) \end{array}$$

Then, the linear velocity and angular velocity of the slip-steering robot are:

(5) $$\{\begin{array}{c} V=\frac{n_{1}r\left(1-\mu_{l}\right)+n_{2}r(1-\mu_{2})}{2}\\ \omega =\frac{n_{2}r\left(1-\mu_{2}\right)-n_{1}r(1-\mu_{1})}{B} \end{array}$$

Suppose the initial position of the robot is *P*_0_ = (*x*_0_, *y*_0_, *θ*_0_), and the pose of the robot after Δ*t* s is *P*_1_ = (*x*_1_, *y*_1_, *θ*_1_), then:

(6) $$\left[\begin{array}{c} x_{1}\\ y_{1}\\ \theta_{1} \end{array}\right]=\left[\begin{array}{c} x_{0}\\ y_{0}\\ \theta_{0} \end{array}\right]+\left[\begin{array}{c} V\Delta t\cdot cos(\theta_{0}+\frac{\omega \Delta t}{2})\\ V\Delta t\cdot sin(\theta_{0}+\frac{\omega \Delta t}{2})\\ \omega \Delta t \end{array}\right]$$

In the simulation, the influence of the slip rate is not considered, we assume that *μ*_l_ = *μ*_r_ = 0.

## 3. Analysis of Four-Wheel Drive Adaptive Robot Algorithm

### 3.1. Introduction to SLAM Algorithms

#### 3.1.1. Gmapping Algorithm

The Gmapping algorithm is a SLAM method based on RBPF (Rao-Blackwellized Particle Filters) [8]. It makes effective use of odometer information and laser scan data and can achieve better a mapping effect in a small indoor environment, so it has been widely used in recent years. RBPF is an algorithm of positioning first and constructing maps later. When the robot observation information *Z* and odometer data *u* are known, the robot’s track *X* and surrounding environment m are solved first, and then the acquired track and observation data are used to construct a map. The joint probability distribution p(*X*_1:*t*_,m|*Z*_1:*t*_,*u*_1:*t*_) can be decomposed into:

(7) $$p\left(X_{1:t},m|Z_{1:t},u_{1:t}\right)=p(m|X_{1:t},Z_{1:t})\cdot p(X_{1:t}|Z_{1:t},u_{1:t})$$

p(m|*X*_1:*t*_,*Z*_1:*t*_) in the formula represents the environment map a posteriori probability, and p(*X*_1:*t*_|*Z*_1:*t*_,*u*_1:*t*_) represents the trajectory of the a posteriori probability.

The key of RBPF algorithm is the solution of p(*X*_1:*t*_|*Z*_1*:t*_,*u*_1:*t*_), which can be estimated by particle filtering. However, since each particle stores the pose, weight, and map of the current moment, memory explosion will occur when the total number of particles N increases. The particle filter inevitably resamples, which leads to particle dissipation, so that the diversity of particles is lost.

The Gmapping algorithm has been improved to address the shortcomings of the RBPF algorithm [9]. The Gmapping algorithm uses maximum likelihood estimation to replace the proposed distribution, and further limits the effective range of the proposed distribution by using the observation data of the latest frame, thus reducing the number of particles to a certain extent. In addition, selective resampling is carried out by setting a threshold, and the threshold *Neff* can be determined by Formula (8).

(8) $$Neff=\frac{1}{\sum_{i-1}^{N}{\left(w^{i}\right)}^{2}}$$

where, *w* is the normalized weight of particle *i*. When *N_eff_* is less than half of the total number of particles, resampling can reduce the sampling times and alleviate particle degradation to some extent.

#### 3.1.2. Hector SLAM

Hector SLAM is a SLAM algorithm based on scanning matching, which ensures the accuracy of raster maps through bilinear interpolation and uses the Gauss–Newton iterative method to solve the optimal matching between scanned data and maps [10] for map construction and location. Hector SLAM does not require odometer data for localization, but relies heavily on high resolution and high scanning frequency lidar.

**Figure 5:**
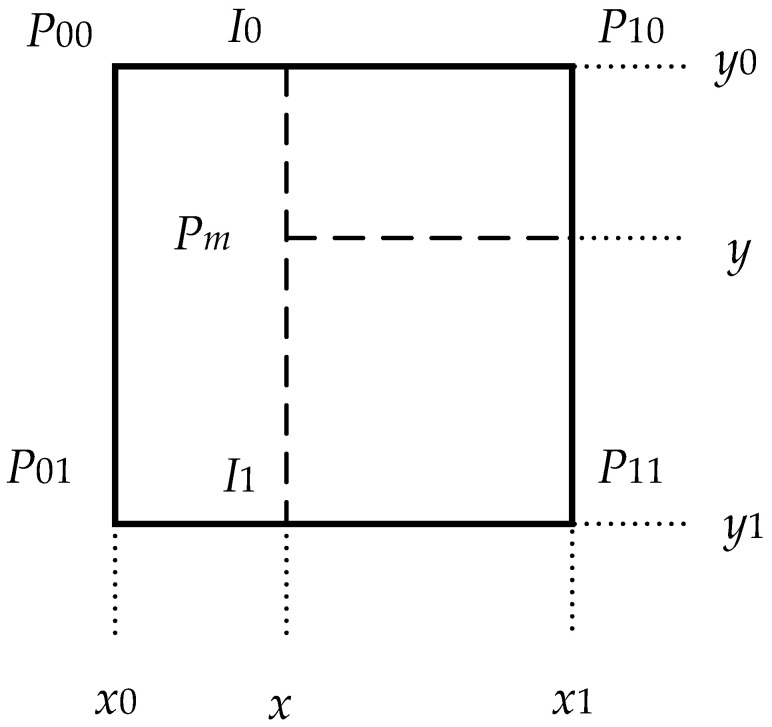
Bilinear interpolation.

In raster maps, the occupancy value *M*(*P_m_*) represents the probability that the continuous coordinate *P_m_* is occupied. As shown in Figure 5, when the four adjacent integer coordinates *P*_00_, *P*_01_, *P*_10_, and *P*_11_ are known, the probability of the *P_m_* point being occupied can be estimated by linear interpolation in the x direction and the y direction, respectively:

(9) $$\begin{array}{l} M(P_{m})\approx \frac{y-y0}{y1-y0}(\frac{x-x0}{x1-x0}M(P_{11})+\frac{x1-x}{x1-x0}M(P_{01}))\\ +\frac{y1-y}{y1-y0}(\frac{x-x0}{x1-x0}M(P_{10})+\frac{x1-x}{x1-x0}M(P_{00})) \end{array}$$

Then the gradient in the *x* and *y* directions can be estimated as:

(10) $$\nabla M(P_{m})=(\frac{\partial M}{\partial x}(P_{m}),\frac{\partial M}{\partial y}(P_{m}))$$

(11) $$\frac{\partial M}{\partial x}(P_{m})\approx \frac{y-y0}{y1-y0}(M(P_{11})-M(P_{01}))+\frac{y1-y}{y1-y0}(M(P_{10})-M(P_{00}))$$

(12) $$\frac{\partial M}{\partial y}(P_{m})\approx \frac{x-x0}{x1-x0}(M(P_{11})-M(P_{01}))+\frac{x1-x}{x1-x0}(M(P_{10})-M(P_{00}))$$

In the actual map building, the first frame data of laser scanning are used to directly build the map. In the subsequent scanning, the obtained data are matched with the existing map, so as to deduce the optimal pose of the robot and update the map.

The assumption in the coordinate system of the robot, laser scanning data coordinate *S_i_* = (*s_i_*, *x*, *s_i_*, *y*)^T^, and corresponding moment robot pose factor *ξ* = (*x*, *y*, *θ*)^T^, *S_i_* can be obtained by polar coordinates transformation in the world coordinates in the coordinate system:

(13) $$S_{i}(\xi )=\left(\begin{array}{cc} \cos(\theta ) & -\sin(\theta )\\ \sin(\theta ) & \cos(\theta ) \end{array}\right)(\frac{s_{i},x}{s_{i},y})+(\frac{x}{y})$$

When scanning and matching, if the alignment error between the laser scanning data and the map reaches the minimum, the robot pose ξ* derived is the optimal pose, that is:

(14) $$\xi^{*}=\arg\min\sum_{i=1}^{m}{\left[1-M(S_{i}(\xi ))\right]}^{2}$$

where, *M*(*S_i_*(*ξ*)) is the corresponding value of *S_i_*(*ξ*).

#### 3.1.3. Karto SLAM

**Figure 6:**
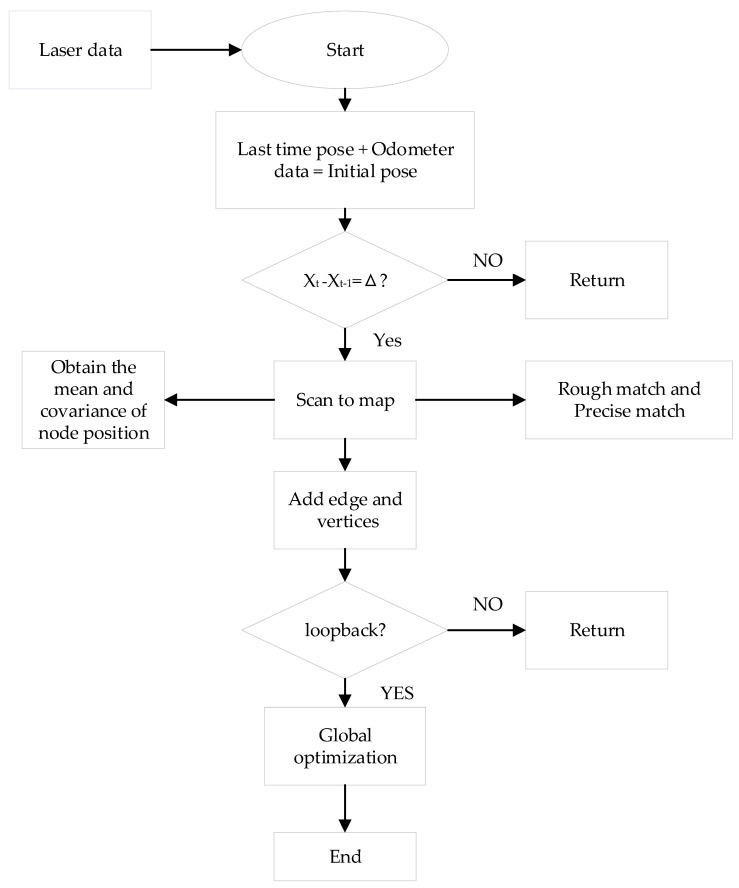
Karto SLAM Flowchart.

The Karto algorithm is a typical SLAM algorithm based on graph optimization [11,12]. Highly optimized and non-iterative Cholesky matrix decompositions are used as the solver of the sparse linear system, and SPA (Sparse Pose Adjustment) is used to complete scan matching and loopback detection [13]. The filtering method has a large storage capacity and has updating efficiency problems. Therefore, it is difficult to be used in scenes with a large map scale [14]. The overall structure of the Karto SLAM algorithm is simple and clear, and the process is shown in Figure 6.

**Figure 7:**
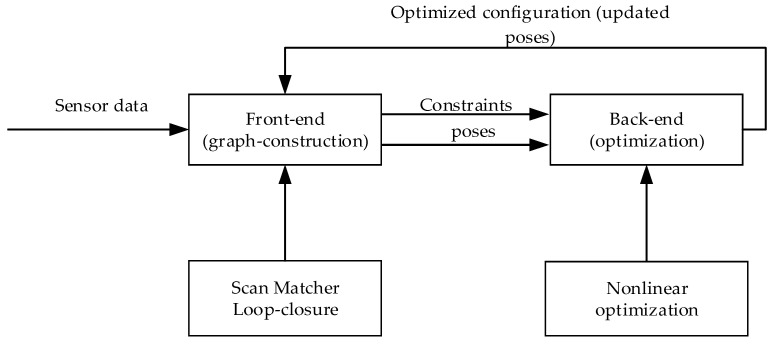
The framework of Karto SLAM.

The Karto SLAM algorithm uses odometer data to assist positioning to get the initial position and pose. On this basis, the lidar scan data are directly matched with the surrounding local map, namely scan-to-map match. For the obtained lidar data, different angles are mapped in the robot’s coordinate system with a certain resolution and an offset value to obtain the lookup table, and the multi-resolution method is adopted to improve the search efficiency. Karto SLAM constructs the front-end graph by scanning and matching the lidar data and loopback detection and transmits constraints and poses to the back end for nonlinear optimization to update poses. The framework of the Karto SLAM is shown in Figure 7.

In the Karto SLAM algorithm, the pose of the key frame is taken as the node, and it is connected with the previous frame, the lidar data link, and the nearest neighbor node to generate edges. During loopback detection, the current frame is extracted to establish a closed-loop connection relationship with adjacent key frames within a certain range, and closed-loop detection is attempted. If closed-loop conditions are formed, it is considered that the current pose of the robot overlaps with the motion track, and then sparse pose adjustment is triggered. The LM optimization algorithm [15] (Levenberg–Marquardt) was used to optimize the global pose, so as to avoid the cumulative error caused by the laser data error and odometer. 

**Figure 8:**
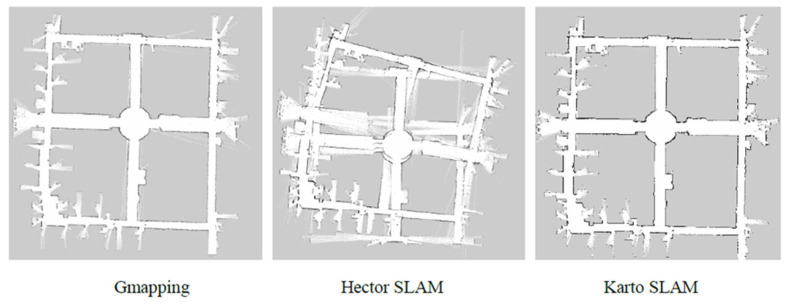
Comparison of three SLAM algorithms.

The data set of the SLAM Benchmark [16] was used to test the three algorithms, and Rviz was used to observe the map drawn by the three algorithms, as shown in Figure 8.

It can be seen that there is little difference between the maps drawn by the Gmapping and Karto SLAM algorithms, while the Hector SLAM algorithm has a relatively poor mapping performance due to the insufficient update frequency and accuracy of lidar used in the dataset, and the lack of loopback detection means. Three SLAM algorithms are compared and summarized as shown in Table 2.

As can be seen from the above table, the Karto SLAM algorithm has relatively low requirements on odometer accuracy and radar frequency, and compared with the filtering method, Karto SLAM adopts graph optimization, with its front-end matching and loopback detection method, which has a higher robustness and a better effect in mapping under the environment.

### 3.2. Introduction to Path Planning Algorithm

#### 3.2.1. Global Path Planning

Global path planning is a static planning method. When the static global cost map is known, the global path planning algorithm can be used to directly calculate the global path from the robot starting point to the target point. In actual navigation, the global path will be refreshed at a certain frequency according to the current position of the robot, so as to avoid navigation errors caused by robot traveling errors or other factors and improve the success rate of navigation to a certain extent.

In the ROS navigation framework, global path planning can be achieved by the Dijkstra and A* algorithms. The Dijkstra algorithm is a graph-based shortest path algorithm [17]. It adopts the width-first search method, expands gradually from the starting point to the target point around, and plans an optimal path. As Dijkstra lacks constraints and has a wide search range in the search process, its algorithm is inefficient. In contrast, the A* algorithm adopts a heuristic search and introduces an evaluation mechanism, which greatly improves the search efficiency [18]. The father–child node relationship is introduced in the A* algorithm, and there are two lists, OPEN and CLOSE. The former stores the nodes to be processed, and the latter stores the nodes that have been processed [19]. In the algorithm search, the evaluation function is expressed as:

(15) f(n)=g(n)+h(n)

where, *n* is the coordinate of the current node, *g*(*n*) is the cost of moving from the initial node *O* to the current node, and the heuristic function *h*(*n*) represents the estimated cost from the current node to the target points. *g*(*n*) can be solved in an iterative way:

(16) $$g(n)=\{\begin{array}{c} dist(O,n)\\ g(p(n))+dist(p(n),n) \end{array}\begin{array}{c} if\\ if \end{array}\begin{array}{c} p(n)=O\\ p(n)\neq O \end{array}$$

*h*(*n*) is usually expressed by the Manhattan distance or the Euclidean distance, the former is less computationally intensive, while the latter is closer to the actual cost.

**Figure 9:**
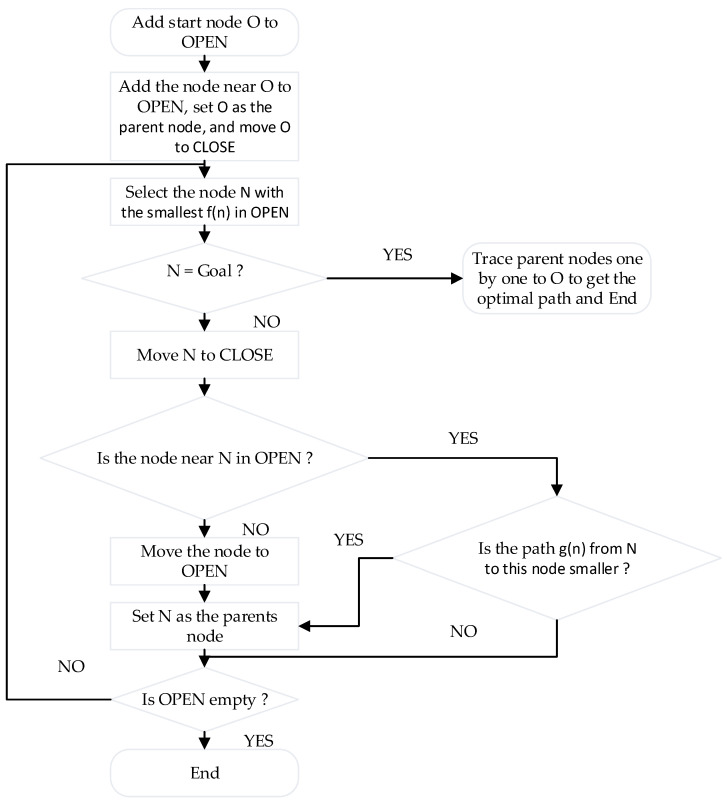
A* Algorithm flowchart.

The algorithm flow of A* is shown in Figure 9.

#### 3.2.2. Local Path Planning

In actual navigation, robots often need to make dynamic decisions according to the surrounding environment to achieve real-time autonomous obstacle avoidance, so as to improve the adaptability to the environment, so local path planning is necessary [20].

In this paper, the DWA (Dynamic Windows Approach) algorithm is used for local obstacle avoidance, and the obstacle avoidance problem is transformed into an optimization problem with constraints in the speed space. Multiple groups of speed samples are obtained by sampling and corresponding trajectories are generated. The trajectory is evaluated by the evaluation function, and the optimal driving strategy can be obtained [21].

## 4. ROS System Simulation of Four-Wheel Drive Adaptive Robot

### 4.1. Establishment of Four-Wheel Drive Adaptive Robot Model

The URDF (Unified Robot Description Format) is an XML file used to describe the Robot model, including the structure, joints, and degrees of freedom of the Robot [22].

In order to realize the simulation of Karto SLAM, it is necessary to simulate lidar, IMU, and other sensors in Gazebo. Add the corresponding plugin to the URDF model, as shown in Table 3.

**Figure 10:**
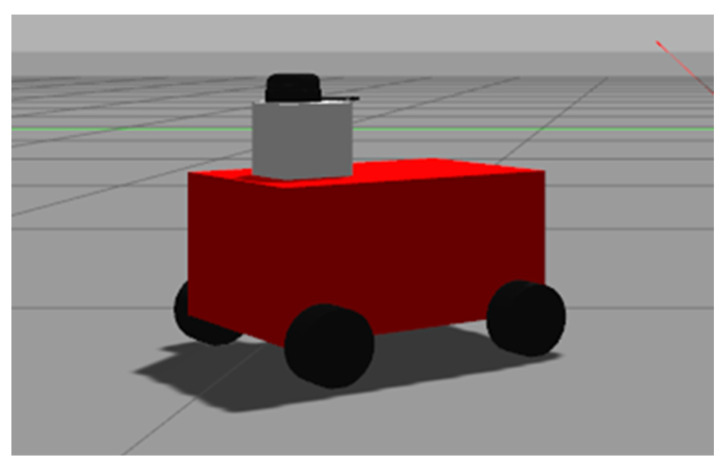
Four-wheel robot model.

The resulting four-wheel robot model is shown in Gazebo in Figure 10.

### 4.2. Mapping Simulation of Four-Wheel Drive Adaptive Robot

**Figure 11:**
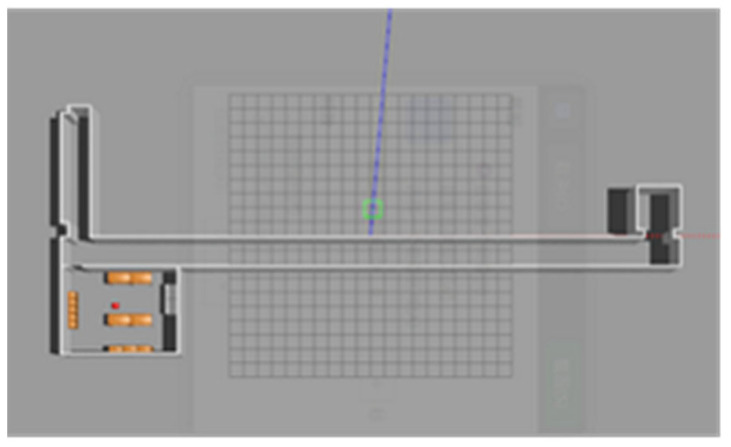
“Lab-corridor” model.

In order to achieve the simulation of map building, we first need to build a simple 3D map model in Gazebo. The simple lab-corridor model created in this paper is shown in Figure 11.

**Figure 12:**
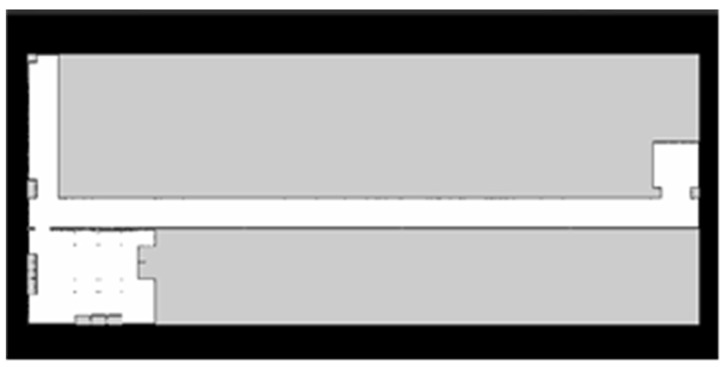
“Lab-corridor” map.

After the map model is created, the simulation of mapping can be started by loading the robot model. The Karto SLAM algorithm is used in the simulation in this paper. Control robots are used to scan the environment and build maps. After scanning, a 2D map is obtained, as shown in Figure 12.

### 4.3. Navigation Simulation of Four-Wheel Drive Adaptive Robot

**Figure 13:**
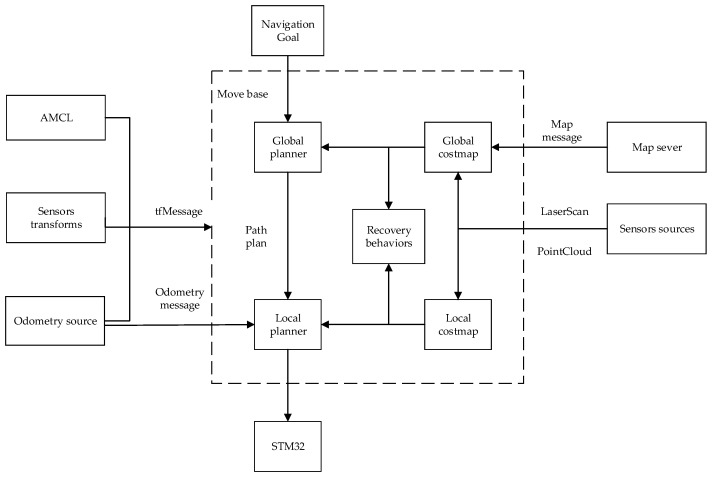
Navigation framework.

The framework of the entire navigation system is shown in Figure 13.

**Figure 14:**
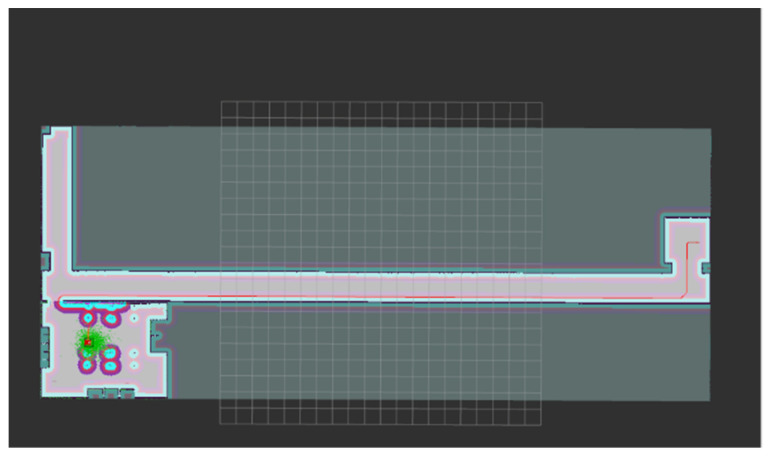
Global path plan.

After relevant parameters are configured, the initial interface of robot navigation is observed in Rviz. The environment of the map is displayed as the global cost map, the environment around the robot is the local cost map, the light blue area is the expansion range of the obstacle, which is the area with the highest possibility of collision and the highest cost, and the purple area is the area with the second highest probability of collision. In the figure, the green point cloud around the robot is the particle distribution in the AMCL positioning algorithm. After setting the target point, the global path planning is shown in Figure 14.

**Figure 15:**
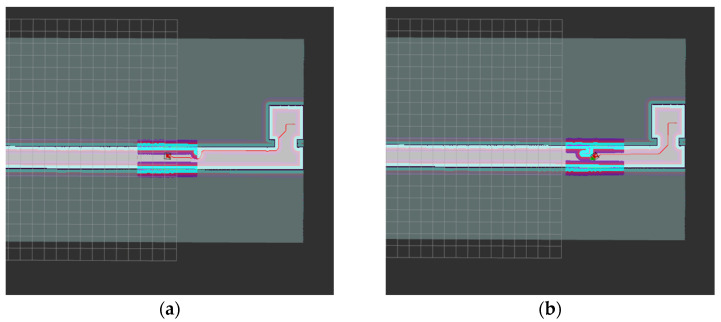
DWA dynamic obstacle avoidance. (**a**) Detect the obstacle; (**b**) Avoid the obstacle.

In the navigation process, new local obstacles are added to the Gazebo physical simulation scene, and the robot dynamically avoids obstacles through the DWA algorithm, as shown in Figure 15.

**Figure 16:**
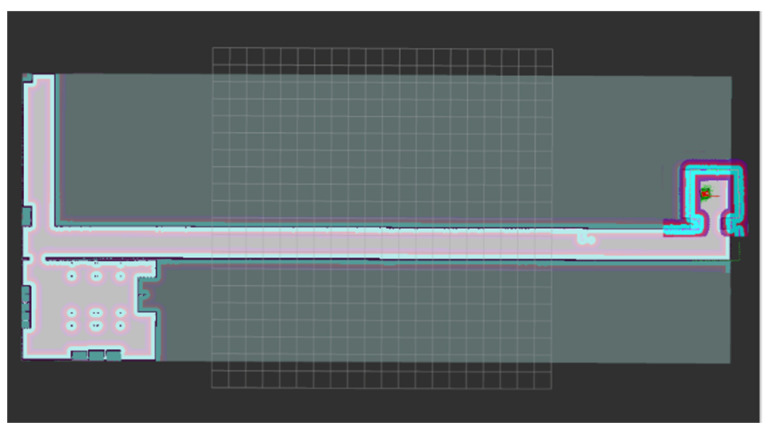
Reach the target point.

After dynamic obstacle avoidance is completed, the robot continues to move along the planned route. Finally, the robot reaches the target point and the navigation ends, as shown in Figure 16.

## 5. Experimental Verification of Four-Wheel Drive Adaptive Robot

### 5.1. Chassis Communication and Function Encapsulation

**Figure 17:**
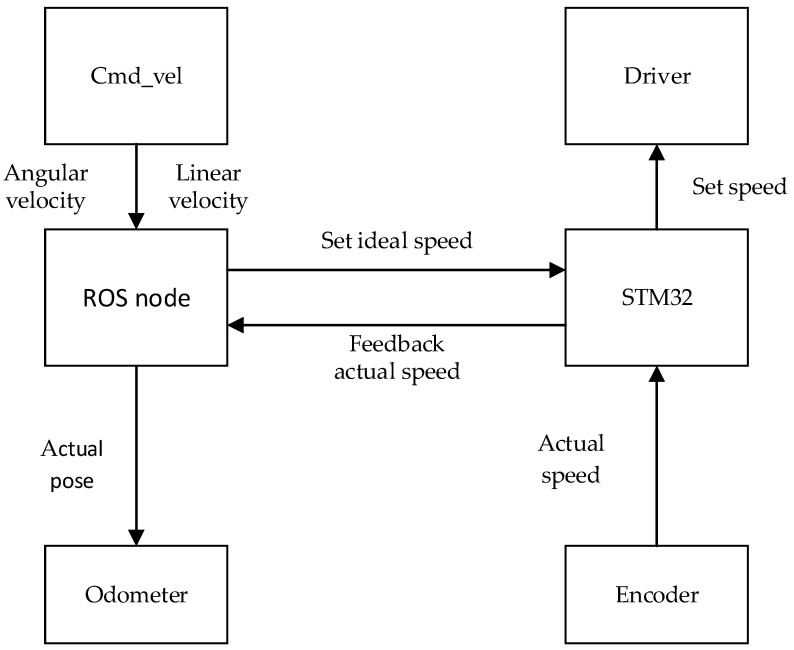
Chassis communication flow.

In this paper, the upper computer is a laptop with an i7-10750H processor and a ubuntu18.04 system installed. The lower computer is a STM32F103 series development board. In the actual navigation, ROS communicates with STM32 through a serial port, and functions are designed to receive speed commands and feedback odometer data. The upper computer releases speed information at a certain frequency, and the lower computer receives this data and controls the chassis to move. At the same time, the lower computer obtains the encoder data as the actual running speed of the robot and feeds it back to the upper computer. After the chassis node obtains the actual speed, it converts it into the actual pose of the robot for odometer use according to the kinematics model. The whole communication process is shown in Figure 17.

Cmd_vel publishes real-time linear and angular velocity topics, the ROS chassis node receives Cmd_vel topics and converts them to the left and right wheel speed. The conversion relationship is as follows:

(17) $$\{\begin{array}{c} V_{l}=v-B\omega /2\\ V_{r}=v+B\omega /2 \end{array}$$

Because there is relative sliding between the wheel and the ground in the steering process of the four-wheel mobile robot, the data of the encoder cannot accurately express the actual speed of the robot, and the conversion relationship between the two exists in the formula above. As for the slip problem of a four-wheel mobile robot, the average slip rate is often used to measure it in practical tests [23]. In this paper, the influence of the slip rate on the actual motion of the robot in in situ steering is mainly considered. At this time, to control the robot chassis to turn *θ* degrees in situ firstly, the actual pose of the robot is *P* = (0, 0, *θ*). Assuming that the slip rate of the wheels on both sides is 0, the mileage pose of the robot *P*’ = (0, 0, *θ*’) can be calculated by the encoder. Since the wheels on both sides are in reverse and the same speed, *μ_l_* = *μ_r_*, then:

(18) $$\theta =\frac{V_{r}\left(1-\mu_{r}\right)-V_{l}(1-\mu_{l})}{B}\cdot \Delta t=\frac{2V_{r}\left(1-\mu_{r}\right)}{B}\cdot \Delta t$$

(19) $$\theta^{\prime }=\frac{2V_{r}}{B}\cdot \Delta t$$

At this point, the slip rate is:

(20) $$\mu_{l}=\mu_{r}=1-\frac{\theta }{\theta^{\prime }}$$

The average slip rate of in situ steering is 0.45 after several experiments.

### 5.2. Mapping and Navigation Experiments

**Figure 18:**
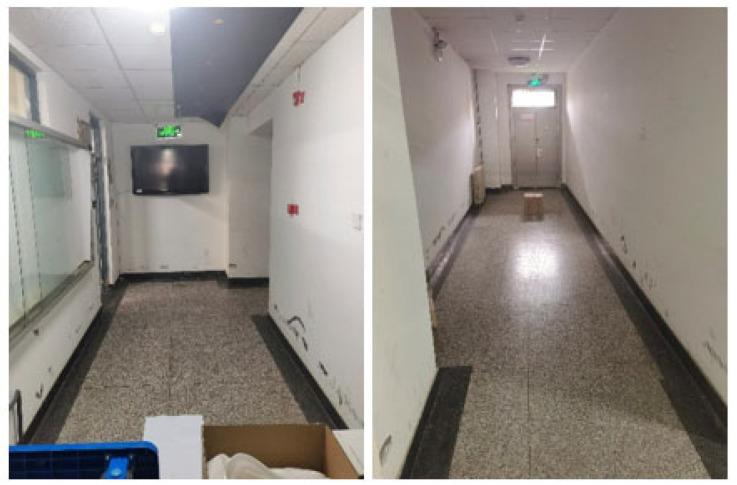
“L” shaped long corridor.

In this paper, the site used in the actual mapping and navigation experiment is the “L” shaped long corridor outside the laboratory. A carton is placed at one end of the corridor to assist in evaluating the mapping accuracy of the mobile robot, and a planar sign with the same size as the robot is placed at the other end of the corridor to evaluate the navigation accuracy of the mobile robot. The environment is shown in Figure 18.

**Figure 19:**
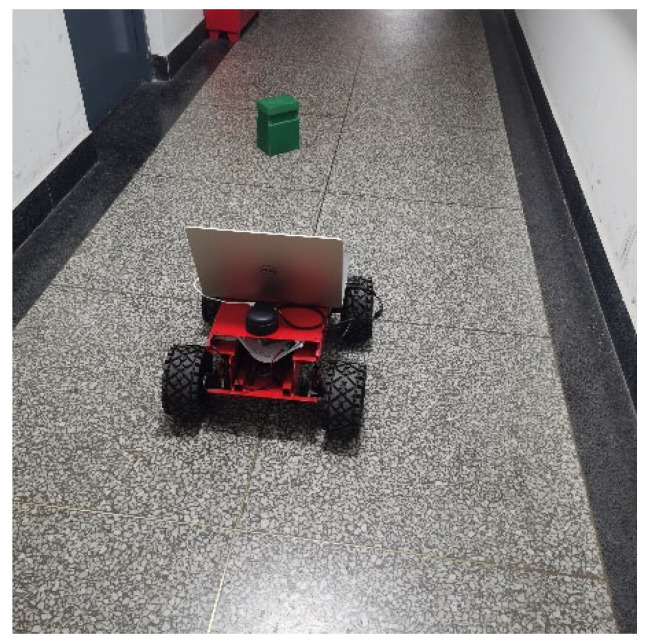
Four-wheel robot.

The robot used in the experiment is shown in Figure 19, which is connected by a chassis, lidar, and upper computer. The lidar is placed on the head of the robot, and the upper computer is placed behind the lidar. During the mapping and navigation experiment, the screen of the upper computer is extended to 180 degrees, so the influence caused by the blocking of the upper computer can be ignored.

**Figure 20:**
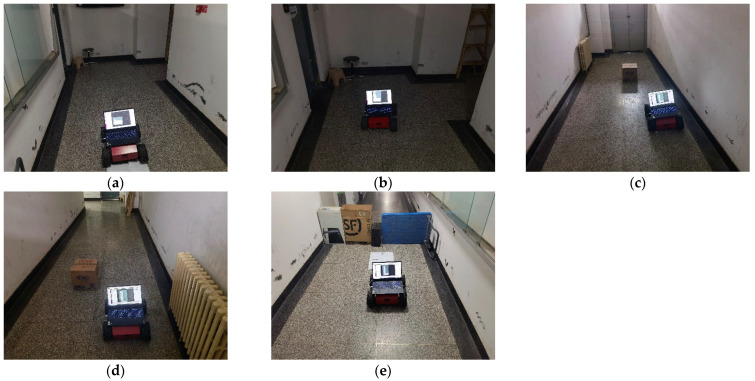
Mapping experiment. (**a**) Start mapping; (**b**) Turn right; (**c**) Avoid the carton; (**d**) Bypass the carton; (**e**) Back to the start point.

The robot is controlled to start from the starting point as shown in the figure below and started to build the map. After controlling the robot to turn right, it bypasses the carton and returns to the starting point to complete the mapping experiment. The whole process is shown in Figure 20.

**Figure 21:**
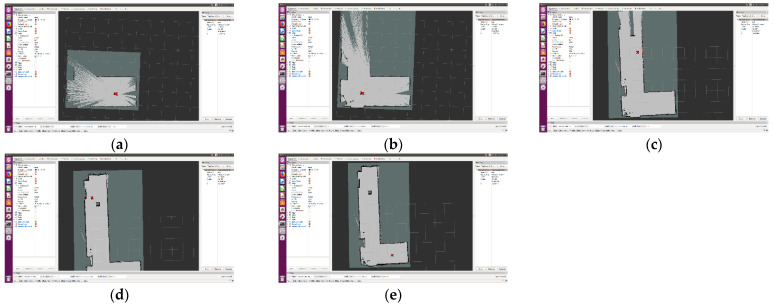
Mapping experiment in Rviz. (**a**) Start mapping; (**b**) Turn right; (**c**) Avoid the carton; (**d**) Bypass the carton; (**e**) Back to the start point.

The mapping experiment process was observed in Rviz as shown in the Figure 21.

**Figure 22:**
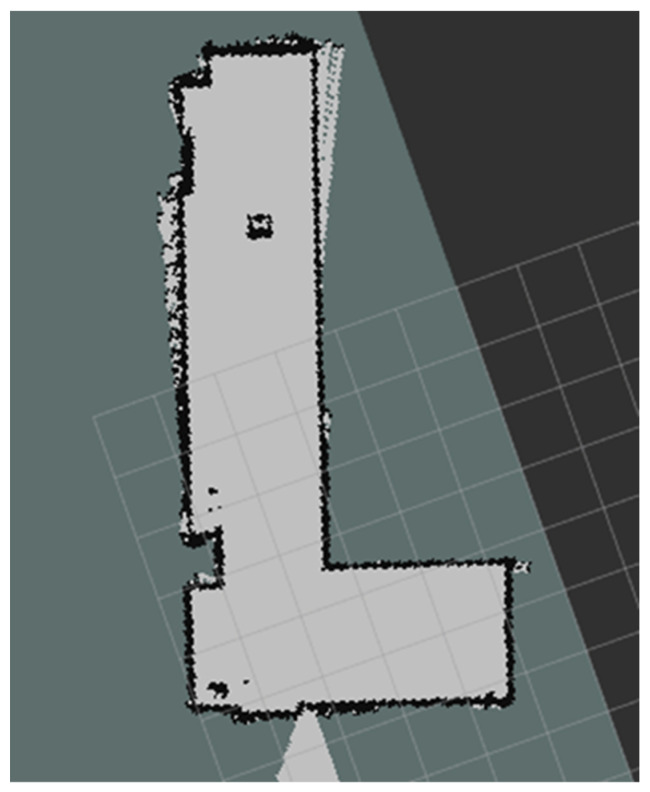
“L” shaped corridor map.

Because the space outside the laboratory is small, the resolution of the map was set at 0.025 m during the map building. Finally, the actual “L” shaped long corridor map obtained by Karto SLAM is shown in Figure 22.

**Figure 23:**
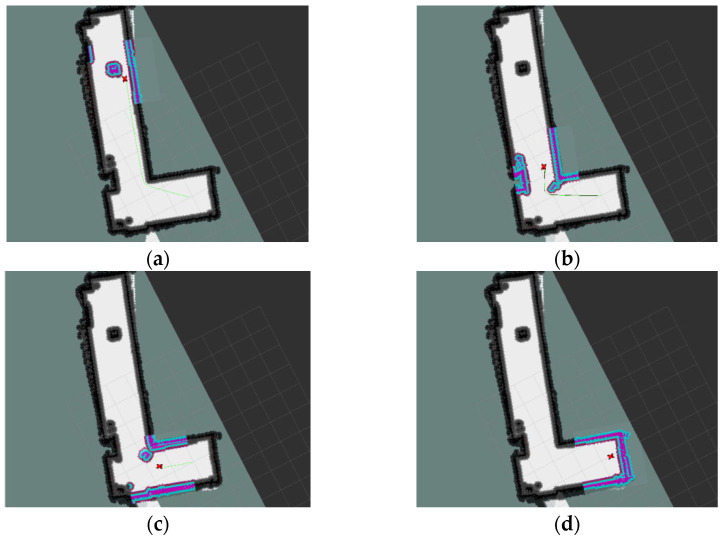
Navigation experiment in Rviz. (**a**) Start navigation; (**b**) Detect the obstacle; (**c**) Avoid the obstacle; (**d**) Reach the target point.

The built map is saved and written to the navigation configuration file. Before the navigation experiment, the robot is placed in the initial navigation position, and the initial pose of the robot model in Rviz is adjusted to be aligned with the actual pose. After setting the target point, it can be seen that the robot uses the A* algorithm to plan the global path and starts moving. The obstacle is placed in the path of the global path, and when the mobile robot detects the obstacle, the DWA is used for local path planning to avoid the obstacle and finally reach the target point. The whole navigation process is observed in Rviz, as shown in Figure 23.

**Figure 24:**
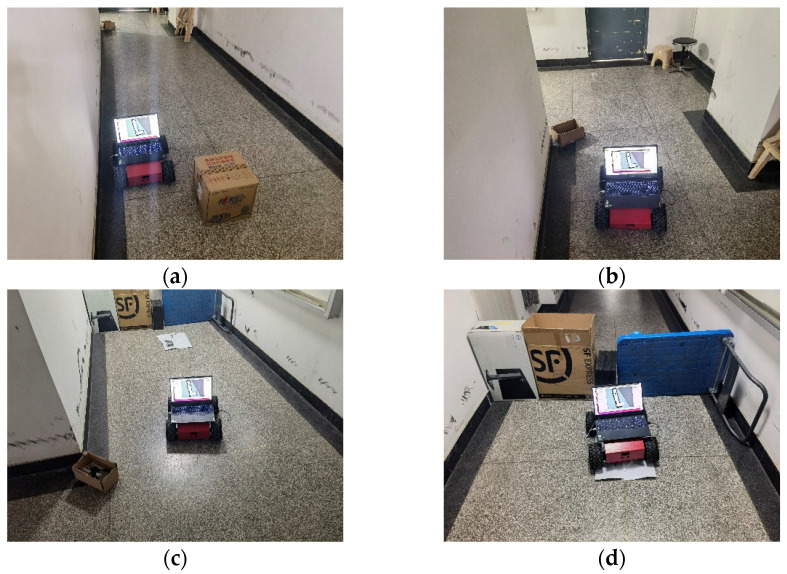
Navigation experiment. (**a**) Start navigation; (**b**) Detect the obstacle; (**c**) Avoid the obstacle; (**d**) Reach the target point.

The navigation process of the actual robot is shown in Figure 24. It can be seen that in the navigation experiment, a new obstacle is added at the turning point of the L-shaped corridor. After detecting the obstacle, the mobile robot adjusts the path through the DWA to avoid the obstacle successfully and reaches the target point.

## 6. System Performance Analysis

### 6.1. Simulation Scene Test

**Figure 25:**
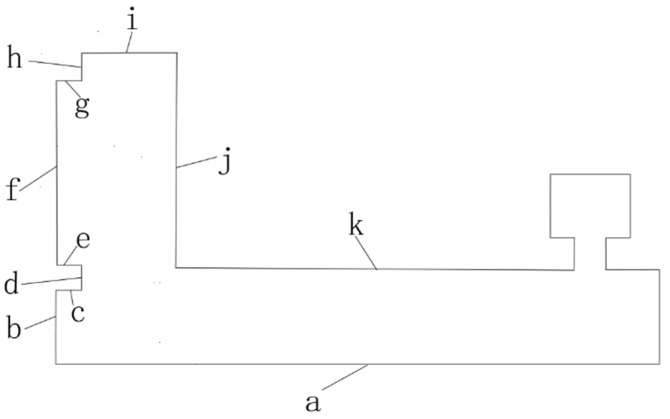
Schematic figure of simulation scene.

In order to quantitatively analyze the mapping accuracy of the system in the simulation scene, several key distances are selected and numbered in the simulation scene. The numbers in the simulation scene are shown in Figure 25.

After the map is built, the measure function in Rviz was used to measure the length of the key distance in the map. The data obtained in multiple experiments are shown in Table 4. The Mean of Measurement is the arithmetic mean of the three measurements. Absolute Error = Mean of Measurement−True value. Relative Error = |Absolute Error|/True value. The numbers in the Table 4 are the same as those in the Figure 25. The Relative Error in the table is reserved for two decimal places, and all remaining values are kept as three decimal places.

**Figure 26:**
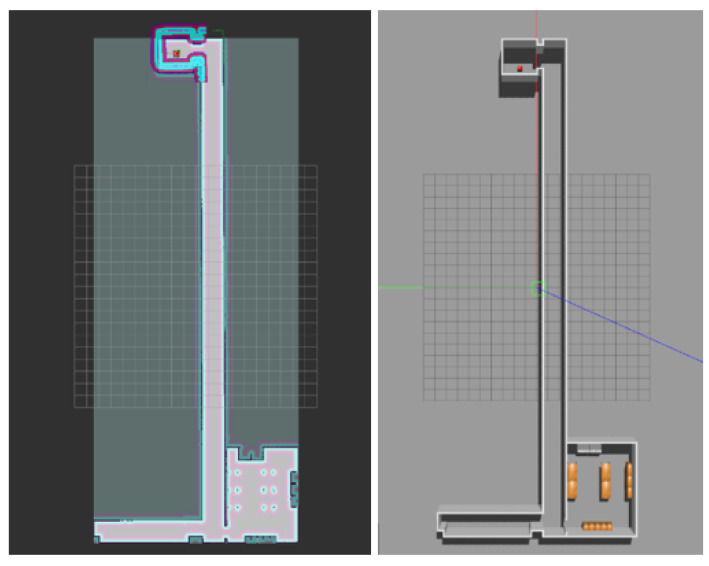
Navigation results of simulation scene.

As can be seen from Table 4, the average map construction accuracy in the simulation scene is 0.158 m, and the average relative error is 1.22%. When the robot arrives at the goal of the navigation, there is no function to measure the distance between the robot model and the wall in Gazebo. The actual navigation accuracy is adopted as the navigation accuracy index. The navigation result in the simulation is shown in the Figure 26.

### 6.2. Actual Scene Test

**Figure 27:**
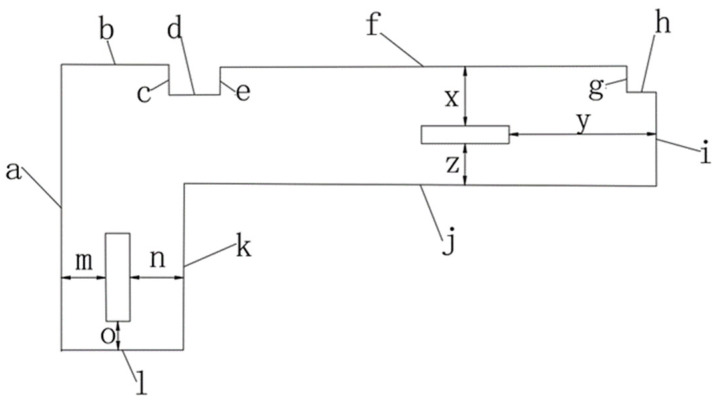
Schematic figure of actual scene.

In order to quantitatively analyze the accuracy of the system mapping and navigation in the actual environment, several key distances are selected in the actual scene as shown in Figure 27.

A laser rangefinder is used to measure the size of the key distance as the true value. The data obtained in multiple experiments are shown in Table 5. When the robot arrives at the goal of the navigation, the deviation of the yaw angle and the distance between the car and the wall are measured to evaluate the navigation accuracy. The Mean of Measurement is the arithmetic mean of the three measurements. Absolute Error = Mean of Measurement−True value. Relative Error = |Absolute Error|/True value. The numbers in Table 5 are the same as those in the Figure 27, the Yaw Angle data *θ* are reserved for one decimal place, the Relative Error in the table is reserved for two decimal places, all remaining values are kept as three decimal places.

As can be seen from Table 5, the average map construction accuracy in the actual scene is 0.019 m, and the average relative error is 1.79%. The actual navigation accuracy is 0.018 m, the relative error is 2.69%, and the angle deviation is 4.3°.

### 6.3. Analysis and Discussion

In this paper, a Karto SLAM algorithm is used for map building. The accuracy of the map construction in a simulation environment is 0.158 m, and the relative error is 1.22%. The accuracy of the map constructed in the actual environment was 0.019 m and the relative error was 1.79%. Compared to Hector and Gmapping, Karto SLAM can avoid lidar data errors and odometer accumulative errors, so the mapping accuracy can be improved. There are some problems such as large computation and a slow update efficiency when using the filtering method to build a map. Compared with the filtering method, Karto SLAM adopts graph optimization with its front-end matching and loopback detection method, which has a higher robustness and a better graph building effect. From the data analysis, it can be seen that the absolute distance error of section a and section k in the mapping simulation is large, which is caused by error accumulation and a lack of loopback detection in the mapping process. The relative error of section i is large, which is because the resolution of the mapping simulation is 0.05 m, and a row of grids cannot be corrected after three experiments of mapping, so there is always an error of about 0.05 m. The relative error of section e, section g, and section h in the mapping experiment is large, reaching 4.69%. The reason for this phenomenon is mainly due to the limitations of the equipment. The map will be offset at the corner, affecting the accuracy of the mapping. With the improvement of the equipment, the accuracy will be closer to the simulation result. Due to the existence of the map expansion coefficient during navigation, the influence of a 4.69% relative error on navigation is acceptable. Due to the influence of human factors in the second experiment, the deviation at section z is too large, resulting in a relative error of 4.57%. If in the second experiment data are discarded, the accuracy will be further improved.

In this paper, A* and DWA algorithms are used for path planning. Compared with Dijkstra’s traversal search method, the A* algorithm uses the evaluation function from the target point to the current point. By estimating the cost to determine the path search direction, the efficiency of the algorithm is improved. The A* algorithm can quickly design the shortest global path without collision in a known environment. The DWA algorithm combines the data of sensors to carry out real-time local path planning, which has a good obstacle avoidance ability and is suitable for robot path planning in a dynamic environment. According to the experimental data, the navigation accuracy of the system is 0.018 m, and the relative error is 2.69%. Part of the reason is the need to manually mark the target point, which is a part of human error. The angle deviation is large, but in practical navigation, the requirement for the head direction when reaching the target point is not high, and the error is within an acceptable range.

Shou Jiaxin chose Hector slam for map building and the Dijkstra algorithm for global path planning, reducing the dependence on the odometer, but there were still problems such as high requirements on lidar frequency and a slow path search efficiency [6]. Wang Peng chose Gmapping for map building and A* and DWA for path planning. By introducing the evaluation function of global information, the problem of falling into a local optimum is solved, but there are still some problems such as a dependence on the odometer and a low precision of map building [5]. Song Kaitai proposed a mobile robot navigation control system based on the integration of laser SLAM localization and real-time obstacle avoidance control [24]. In his paper, autonomous navigation motion control of the robot was accomplished by combining target navigation with a real-time obstacle avoidance controller. Using the safety weight parameter design, the target seeking control was combined with real-time obstacle avoidance to obtain a safe and accurate guidance control. It improved the security in the process of target searching and map building, but still had the problem of large computation and a dependence on IMU. Andrey proposed a safe map building and area search algorithm for a mobile robot in a closed unknown environment with obstacles [25]. The proposed algorithm was based on a 2D range finder sensor, which makes the data processing simpler than the most of frontier-based algorithms. His mathematical analysis was very rigorous, but his data analysis of the experimental results was simple. Chen Yongbo proposed an active SLAM framework for collision-free trajectory and area coverage for mobile robots using a predictive control model [26]. In his paper, a submap joining method was applied to ensure the effectiveness of the proposed method and improve its runtime performance. A graph topology and convex optimization approach were used to solve the *D-opt* MPC problem and the SQP method was used to solve the coverage problem. Different from traditional path planning, active SLAM involves estimating the location of the robot as it moves and can consider environments that are fully or partially unknown. However, many existing online coverage methods lead to an abrupt velocity and orientation changes when encountering obstacles. This effect becomes pronounced especially when operating in an obstacle-cluttered environments, the success rate of area coverage and map building is sensitive to the quality of sensor input [27]. Meanwhile, performance, safety, and efficiency are the main issues that must be addressed, especially in real-time problems and unknown environments. In this paper, we solve this problem with two separate processes: map building and path planning. A Karto SLAM algorithm is used for map building. Due to Karto SLAM’s advantages of low lidar frequency requirements, front-end matching, and loopback detection, the mapping accuracy is high and the effect is good. A* and DWA algorithms are used for path planning. The A* algorithm introduces the cost function from the target point to the current point and determines the path search direction by estimating the cost. The DWA algorithm combines sensor data for real-time local path planning, which is suitable for robot path planning in a dynamic environment.

## 7. Conclusions

A four-wheel drive adaptive robot mapping and navigation system based on ROS is proposed. In this paper, the Karto SLAM algorithm is selected to construct a 2D map after comparing the mapping effects of various 2D laser SLAM algorithms. The A* algorithm is used to carry out global force planning to optimize the global path for robot planning. The DWA algorithm is used to carry out local path planning to achieve real-time obstacle avoidance. Speed instructions are issued by the move_base package. The communication between ROS and STM32 is realized, and the chassis node is encapsulated, so that it can receive the actual speed command and feedback odometer data. The design function of the lower computer can receive the expected speed and convert it into wheel speed and return the speed feedback from the encoder. The slip rate of the four-wheel robot in situ steering is calculated successfully, which makes the chassis pose more accurate. After the simulation verifies the rationality of the navigation system, the 2D map of the corridor outside the laboratory is completed in the actual test. After setting the target point, the robot can move to the target point independently and achieve navigation successfully.

## Figures and Tables

**Table 1:** The main components and models of the robot.

Module	Model
Chassis	Four-wheel differential chassis
Motor	Faulhaber Dc servo motor
Motor driver	Rmds-108 DC servo motor driver
Controller	STM32F103
Lidar	Rplidar_A2

**Table 2:** SLAM Algorithm comparison.

Algorithm Name	Gmapping	Hector SLAM	Karto SLAM
Principle	Filter	Optimization	Graph optimization
Lidar frequency requirement	Low	High	Low
Odometer accuracy requirement	High	No	Low
Loopback	No	No	Yes
Robustness	High	Low	High

**Table 3:** Plugin description in Gazebo.

The Plugin Name	Description
libgazebo_ros_skid_steer_drive.so	Sliding steering motion plugin
libgazebo_ros_imu.so	IMU sensor plugin
libgazebo_ros_laser.so	Lader plugin
Libgazebo_ros_skid_steer_drive.so	Sliding steering motion plugin
libgazebo_ros_imu.so	IMU sensor plugin
libgazebo_ros_laser.so	Lader plugin

**Table 4:** Mapping data in simulation scene.

Number	True Value (m)	Measurement 1 (m)	Measurement 2 (m)	Measurement 3 (m)	Mean of Measurements (m)	Absolute Error (m)	Relative Error (%)
a	41.000	40.287	40.036	40.266	40.196	−0.804	2.01
b	1.850	1.796	1.840	1.842	1.826	−0.024	1.30
c	0.500	0.498	0.496	0.497	0.497	−0.003	0.60
d	1.150	1.167	1.157	1.156	1.160	0.010	0.87
e	0.500	0.497	0.496	0.498	0.497	−0.003	0.60
f	7.350	7.279	7.334	7.332	7.315	−0.035	0.48
g	0.500	0.498	0.495	0.496	0.496	−0.004	0.80
h	0.500	0.502	0.494	0.498	0.498	−0.002	0.40
i	1.350	1.293	1.295	1.297	1.295	−0.055	4.07
j	9.000	8.996	8.946	8.998	8.980	−0.020	0.22
k	38.150	37.500	37.277	37.335	37.371	−0.779	2.04

**Table 5:** Plugin description in Gazebo.

Number	True Value (m)	Measurement 1 (m)	Measurement 2 (m)	Measurement 3 (m)	Mean of Measurements (m)	Absolute Error (m)	Relative Error (%)
a	4.900	4.877	4.882	4.886	4.882	−0.018	0.37
b	1.830	1.902	1.780	1.840	1.841	0.011	0.60
c	0.515	0.546	0.502	0.508	0.519	0.004	0.78
d	0.870	0.885	0.890	0.876	0.884	0.014	1.61
e	0.470	0.510	0.478	0.476	0.488	0.018	3.83
f	6.930	6.898	6.895	6.902	6.898	−0.032	0.46
g	0.445	0.510	0.452	0.456	0.473	0.018	4.04
h	0.500	0.428	0.484	0.495	0.469	−0.031	6.20
i	1.600	1.582	1.586	1.591	1.586	−0.014	0.88
j	8.050	7.982	8.020	8.032	8.011	−0.039	0.48
k	2.840	2.795	2.828	2.832	2.818	−0.022	0.77
l	2.080	2.020	2.090	2.092	2.067	−0.013	0.63
x	1.015	1.004	1.005	1.007	1.005	−0.010	0.99
y	2.500	2.478	2.486	2.488	2.484	−0.016	0.64
z	0.700	0.674	0.829	0.683	0.732	0.032	4.57
m	0.750	0.735	0.738	0.736	0.736	−0.014	1.87
n	0.910	0.922	0.934	0.937	0.931	0.021	2.31
o	0.490	0.470	0.476	0.468	0.471	−0.019	3.88
θ	0°	15.0°	6.0°	−8.0°	4.3°	4.3°	

## Data Availability

The data are available upon request.
